# Identification of Atypical Peri-Nuclear Multivesicular Bodies in Oxidative and Glycolytic Skeletal Muscle of Aged and Pompe's Disease Mouse Models

**DOI:** 10.3389/fphys.2015.00393

**Published:** 2015-12-21

**Authors:** Brian A. Neel, Haihong Zong, Jonathan M. Backer, Jeffrey E. Pessin

**Affiliations:** ^1^Department of Medicine, Price Center for Genetic and Translational Medicine, Albert Einstein College of MedicineBronx, NY, USA; ^2^Department of Molecular Pharmacology, Albert Einstein College of MedicineBronx, NY, USA; ^3^Department of Biochemistry, Albert Einstein College of MedicineNew York, NY, USA

**Keywords:** multivesicular bodies, lipofuscin, skeletal muscle, sarcopenia, Pompe's Disease

## Abstract

Muscle wasting that occurs during aging or from disease pathology presents with an accumulation of lipid species termed ceroid or lipofuscin. This unique species of lipid has been characterized in various cell types but its properties and organization in skeletal muscle remains unclear. Using immunofluorescence and transmission electron microscopy, we were able to visualize and characterize an atypical lipid storing organelle in skeletal muscle. White myofibers contain two organelles at each pole of the myonuclei and red myofibers contain many of these structures in and around the perinuclear space. These organelles contain markers for late endosomes, are morphologically similar to multivesicular bodies, store lipid, and hypertrophy in aged muscle and a model of muscle wasting with an accumulation of large amounts of lipofuscin. Rapamycin treatment reduces the multivesicular body hypertrophy, restores late endosomal protein markers, and also increases the number and intensity of lipofuscin deposits. Together, these data demonstrate for the first time a perinuclear organelle in skeletal muscle that hypertrophies in muscle wasting phenotypes and is involved in endocytic lipid storage.

## Introduction

The late endosome, or multivesicular body (MVB), was originally characterized as a unique endosomal compartment in the 1950s (Palade, 1955; Sotelo and Porter, 1959). The membrane bound organelle contains many intraluminal vesicles (ILVs) that encapsulate cargo destined for degradation via the lysosome. These ILVs are the result of the endosomal sorting complex required for transport (ESCRT) protein machinery. This protein complex responds to signals like ubiquitination and packages cytoplasmic proteins into the ILVs. These ILVs are also enriched in specialized membrane proteins and unique lipid species (Hirst et al., 1998; Matsuo et al., 2004). Upon maturation, the MVB fuses and releases the ILVs into the acidic lumen of the lysosome, which are then degraded by the numerous hydrolases present. This unidirectional process makes the MVB the last sorting step for cargo in the endocytic pathway (Piper and Katzmann, 2007; Hanson and Cashikar, 2012). Cross talk with the autophagic pathway has also been demonstrated as MVBs can fuse with autophagasomes, creating hybrid organelles called amphisomes (Filimonenko et al., 2007). Recent findings have also demonstrated that the cargo in the ILVs can be released into the interstitial space as structures termed exosomes, which arise by the fusion of the MVBs with the plasma membrane (Raposo and Stoorvogel, 2013).

Skeletal muscle is one of the three types of muscle, and functions to support the skeleton and facilitate movement by connecting to the bones via tendons. Skeletal muscle has a unique architecture to enable its function of contraction and relaxation. The individual muscle cell, or myofiber, is an elongated, multinucleated, post-mitotic cell resulting from the fusion of hundreds of muscle precursor cells. Each myofiber contains many contractile units called sarcomeres, which contain the actin filaments and myosin motors that mechanically undergo contraction and relaxation. Individual myofibers exist in larger bundles called a fascicle that encompasses roughly 100 myofibers contained within a sheath of connective tissue called the epimysium. Many fascicles then make up the larger architecture of the muscle, all contained within a second sheath of connective tissue called the perimysium. Innervation of the muscle combined with calcium flux from the sarcoplasmic reticulum allows all the individual myofibers to contract in unison to produce force (Au, 2004; Braun and Gautel, 2011).

Skeletal muscle can be classified into multiple fiber subtypes based upon expression of various myosin isoforms, rates of contraction, and metabolic fuels (glucose and fatty acids) used as energy sources. In general, the two major fiber types are red, oxidative, type I fibers and white, glycolytic, type II fibers. Red muscle is high in myoglobin and mitochondria, primarily undergoes oxidative metabolism, and is resistant to fatigue. White muscle is relatively low in myoglobin and mitochondria, primarily undergoes glycolytic metabolism, and is susceptible to fatigue. Numerous research articles and reviews have detailed many differences between the fiber types, including differential sensitivity to extracellular signals and specificity for muscle wasting in certain diseases (Schiaffino and Reggiani, 2011; Wang and Pessin, 2013). Skeletal muscle wasting is generally defined by loss of muscle mass and strength and can be caused by a multitude of factors from diseases, drugs, denervation, disuse, and aging.

Much work has been done examining the loss of muscle mass and function in relation to aging, a physiologic process termed sarcopenia. Although it is unclear why aged muscle wastes, it has been clearly demonstrated that this is part of the normal progression of aging. Research has shown that satellite cell number and activation decreases, skeletal muscle regeneration rates decrease, apoptosis increases, and physiological processes like protein synthesis and autophagic and lysosomal degradation become compromised in aged muscle (Dirks and Leeuwenburgh, 2002; Marzetti and Leeuwenburgh, 2006; Shefer et al., 2006, 2010; Wohlgemuth et al., 2010; Chakkalakal et al., 2012). Although these studies are important in relation to investigating aged skeletal muscle biology, they do not address whether these processes are the cause or response to the development of sarcopenia.

Interestingly, other forms of muscle wasting caused by specific diseases also present with many of the same cellular phenotypes seen in aging. For example, Pompe's Disease is a glycogen storage disorder where large amounts of glycogen accumulate specifically in skeletal and cardiac muscle. This is due to a mutation in alpha acid glucosidase (GAA) which functions to break down glycogen into glucose in the lysosome. The adult onset phenotype presents with severe peripheral muscle wasting that is primarily reserved to white muscle.

The accumulation of an inert lipid byproduct called lipofuscin is common to both sarcopenia and Pompe's Disease and results from reduced lysosomal degradation (Hütter et al., 2007; Feeney et al., 2014). Metabolic byproducts build up and become oxidized to create a material that contains fatty acids, proteins, sugars, and metals that cannot be degraded or exocytosed (Brunk and Terman, 2002; Terman and Brunk, 2004; Jung et al., 2007). Lipofuscin has been well characterized to be a hallmark of aging that accumulates in post-mitotic cells, primarily in perinuclear pools (Nakae et al., 2004). Lipofuscin has also been implicated in the pathologies of many diseases such as Alzheimer's, Parkinson's, amyotrophic lateral sclerosis, and lysosomal storage disorders (D'Andrea et al., 2002; Hesselink et al., 2003; Zheng et al., 2010; Bandyopadhyay et al., 2014). Despite many studies examining lipofuscin, many basic questions remain as to how it accumulates, whether it is pathogenic, and whether it can be cleared.

Very few drugs or therapies have been shown to be effective to combat the loss of muscle mass and strength seen in sarcopenia and Pompe's Disease. Rapamycin is a naturally occurring bacterial byproduct that has been shown to be an inhibitor of the protein kinase, target of rapamycin (TOR; Ballou and Lin, 2008). In mammalian cells, the mTOR complex 1 (mTORC1) senses nutrient availability, stress, growth factors, oxygen abundance, and DNA damage, and controls downstream processes like protein synthesis, autophagy, energy metabolism, lipid biosynthesis, and lysosome biogenesis (Laplante and Sabatini, 2009). Through mechanisms not completely defined, rapamycin has been shown to promote longevity in a variety of species. Rapamycin modulates skeletal muscle mass as well as specific metabolic functions, including glucose and lipid utilization. However, its effect on sarcopenia is still not conclusive, with some research suggesting the effect of rapamycin on the loss of muscle mass and strength is minimal (Sipula et al., 2006; Ballou and Lin, 2008; Neff et al., 2013; Zhang et al., 2014). Nonetheless, rapamycin has a beneficial effect on Pompe's Disease by promoting clearance of the excess glycogen (Ashe et al., 2010).

In this study, we have identified a perinuclear MVB-like endocytic compartment that functions to sequester lipofuscin. These unusual MVBs store lipofuscin but have a fiber type-specific localization in different myofibers. In aged and Pompe skeletal muscle, we show that these compartments specifically enlarge as compared to other peripheral compartments and that they accumulate lipofuscin. Rapamycin treatment can reverse the hypertrophy of this compartment seen in sarcopenic muscle. To our knowledge this is the first study showing that lipofuscin is contained within a perinuclear multivesicular body like compartment in a fiber type specific manner.

## Methods

### Animals

All animal experiments received ethical approval and were performed in agreement with the Albert Einstein College of Medicine Institutional Animal Care and Use Committee (2013-1010). Young 2 month old C57Bl/6J mice were obtained from Jackson Laboratories. Aged C57Bl/6J mice (~24 months) were obtained from the National Institute of Aging. 2 month old GAA-deficient Pompe mice (6^neo^/6^neo^) on the C57Bl/6J background, as described by Raben et al. (1998), were provided by Dr. Steven Walkley (Albert Einstein College of Medicine). Mice were housed in a facility equipped with a 12 h light/dark cycle with free access to food and water and were fed a normal chow diet (Research Diets).

### Single myofiber isolation

Mice were sacrificed and the extensor digitorum longus (EDL) and soleus muscles were dissected from the proximal to distal tendon. The muscles were then digested in 0.2% type I collagenase (Sigma, St. Louis MO, USA) in a 37°C water bath with gentle agitation for 60–90 min. Individual myofibers were harvested by pipetting media over the muscle until the fibers dissociated. Viable fibers were assessed as single, non-clumped myofibers, elongated and not contracted, and intact without any apparent cuts, bends, or wounds. The EDL muscle digests faster and separation of the myofibers is easier and the yield of viable myofibers is ~80–90%. The soleus takes longer to digest and the red myofibers are more easily torn and destroyed. A good digestion may yield about half the fibers viable. These viable fibers were then transferred into Dulbecco's Modified Eagle Media (Fisher Scientific, Pittsburg, PA, USA) until the start of the respective assay.

### Immunofluorescence

Individual myofibers were isolated as described above and then transferred to a 4% paraformaldehyde solution (Electron Microscopy Sciences, Hatfield, PA, USA) and fixed at 37°C for 30 min followed by two washes in PBS. The fixed fibers were transferred to a blocking/permeabilization buffer for 2 h [10% horse serum (Fisher Scientific, Pittsburg, PA, USA), 0.1% Triton X100, 10 mg/ml bovine serum albumin in PBS]. Myofibers were incubated in primary antibodies overnight at 4°C with gentle rocking. After repeated washings in PBS, the myofibers were incubated in fluorescent secondary antibodies (Life Technologies, Eugene OR, USA) for 2 h at room temperature with gentle rocking. After repeated washes in PBS, the fibers were mounted on a coverslip with anti-fade Dapi mounting medium (Cell Signaling, Danvers MA, USA). The following primary antibodies were used: Lamp1 (BD Pharmigen, San Jose, CA, USA), M6PR (Abcam, Cambridge, MA, USA), LBPA (Echelon Biosciences, Salt Lake City, UT, USA). Samples were imaged using a Leica SP5 AOBS confocal microscope using a 63x objective. Image analysis was completed using the Lecia LAS-AF software and ImageJ. For each immunostaining sample, at least 20 fibers were analyzed and for each condition, at least three muscles were examined from different mice.

### Transmission electron microscopy

Mice were sacrificed and the EDL and soleus muscles were dissected from the proximal to distal tendon. The muscles were then finely cut lengthwise into ~10 pieces with a razor and fixed with 2.5% glutaraldehyde, 2% paraformaldehyde in 0.1 M sodium cacodylate buffer, postfixed with 1% osmium tetroxide followed by 2% uranyl acetate, dehydrated through a graded series of ethanol and embedded in LX112 resin (LADD Research Industries, Burlington, VT, USA). Ultrathin sections were cut on a Reichert Ultracut UCT, stained with uranyl acetate followed by lead citrate and viewed on a JEOL 1200EX transmission electron microscope at 80 kv.

### Focused ion beam scanning electron microscopy (FIB/SEM)

Individual myofibers from the EDL were harvested and fixed in glutaraldehyde as described above. Staining and Durcupan ACM resin embedding (EMS) was performed as described (Deerinck et al., 2010). The sample block was then mounted on a SEM sample holder using double sided carbon tape (EMS, Hatfield, PA). Colloidal silver paint (EMS) was used to electrically ground the exposed edges of the tissue block and the entire surface of the specimen was then sputter coated with a thin layer of gold/palladium (Denton Desk V). The tissue was imaged using back-scattered electron (BSE) mode in a FEI Helios Nanolab 650. Images were recorded after each round of ion beam milling using the SEM beam at 2 keV and 100 pA with a working distance of 4 mm. Data acquisition occurred in an automated way using the Auto Slice and View G3 software, with an XY pixel size of 11–13 nm and Z step size of 25 nm, resulting in typical volumes of 41.5 μm by 44 μm by 24.5 μm.

### Processing of SEM image stacks

Raw SEM images were cropped, and aligned using the image processing programs in IMOD (UC Boulder). In particular, the stack of SEM images were aligned using IMOD's xfalign and Midas programs. Further image analysis utilized a polynomial filter in Image J (NIH) to reduce an overall intensity gradient. Finally, the images were binned to produce isotropic voxels in the x, y, and z direction (26.5 nm) with Amira software (Visage Imaging, Andover, MA).

### Oil red O and lipofuscin immunofluorescence

Individual myofibers were harvested and fixed as described above. Immunostaining, described above, was performed first, followed by oil red O (ORO) staining (Koopman et al., 2001). A 5 mg/ml stock solution of ORO was made in 60% triethyl-phosphate. Prior to use, a 37% working solution was made and filtered twice or until the solution is completely clear. ORO was added to the previously immunostained myofibers for 30 min at room temperature with gentle rocking. The fibers were then washed numerous times with H_2_O until all leftover ORO was removed and then the fibers were mounted as described above. ORO can be visualized by immunofluorescence at 594 nm. For lipofuscin imaging, myofibers were mounted and visualized at 488 and 594 nm directly after fixation without any staining.

### Rapamycin studies

Male C57Bl/6j mice aged ~24 and ~2 months were treated with rapamycin or vehicle. Rapamcyin was prepared as a 1 mg/ml stock in 10% PEG400/10% Tween80 in ethanol. Mice underwent a daily intraperitoneal injection at a concentration of 4 mg/kg of body weight for 1 month. Mice were then sacrificed and samples were harvested, processed, and imaged as described above.

### Immunoblotting

Skeletal muscle extracts were homogenized in ice-cold lysis buffer (50 mM Tris, pH 7.5, 150 mM NaCl, 1% Triton X-100, 1 mM EDTA, 1 mM phenylmethylsulfonyl fluoride, 0.25% sodium deoxycholate, 1 mM NaF, 1 mM Na_3_VO_4_, and 2 mM Na_4_P_2_O_7_) containing a protease inhibitor mixture (Roche Diagnostics, Indianapolis, IN, USA). The resultant lysates were centrifuged at 16,000 × *g* for 10 min at 4°C, and protein concentrations were quantified using the BCA (bicinchoninic acid) protein assays (ThermoFisher, Grand Island, NY, USA). The protein samples (30 μg) were separated on a 4–12% gradient SDS-PAGE gel and transferred to nitrocellulose membranes using a semidry electroblotter (Owl Separation System, Portsmouth, NH, USA). Membranes were immunoblotted with Lamp1 and GAPDH antibodies (Abcam, Cambridge, MA, USA). Quantification of all immunoblots was performed using NIH IMAGE software.

### Statistical analysis

Two tailed unpaired student's *t*-test was used to evaluate statistical significance in all quantitation analyses. Data are presented as a standard deviation of the mean and *p* < 0.5 (^*^) was considered significant.

## Results

### Skeletal muscle has a unique perinuclear organelle

**Figure 1:**
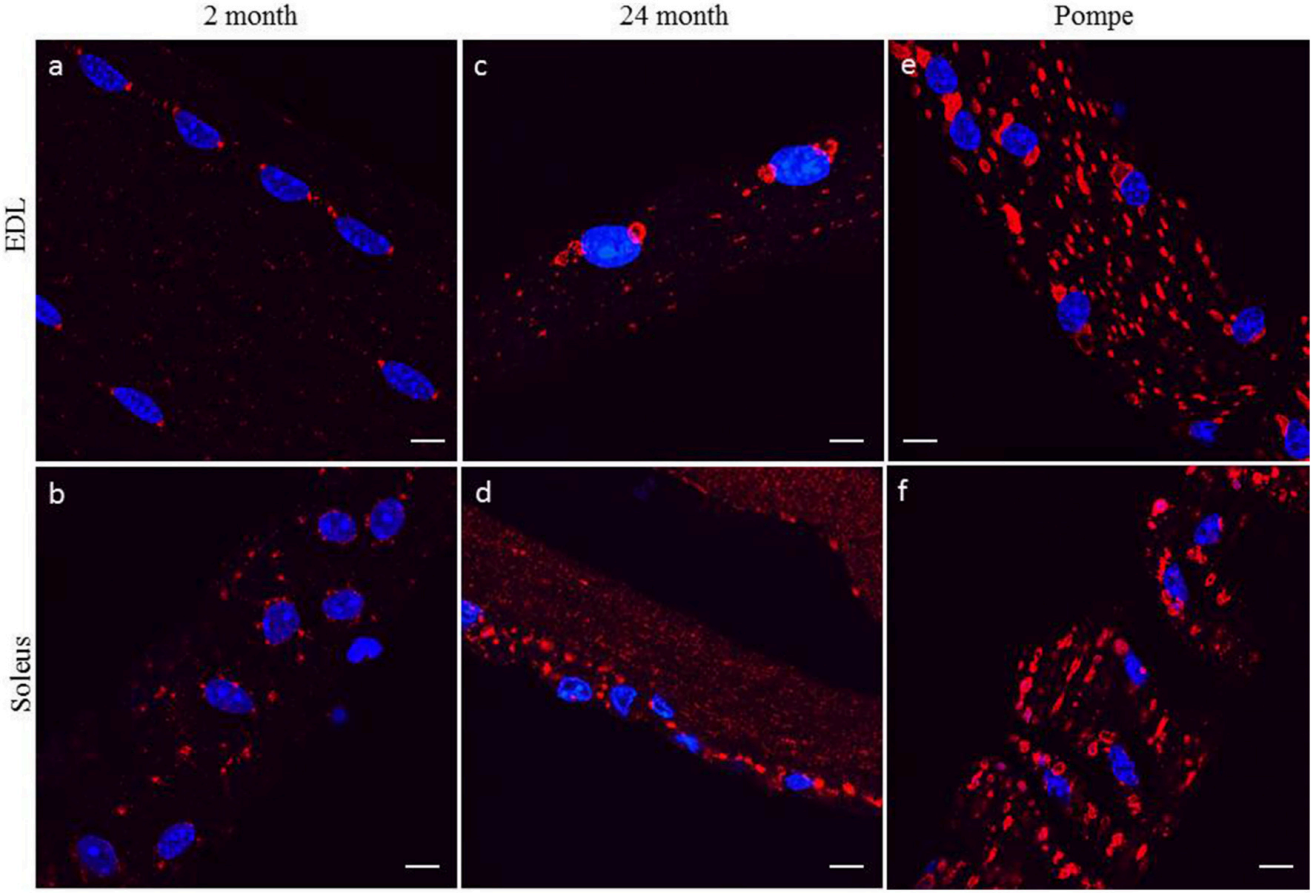
**Individual muscles were digested and myofibers harvested from EDL or soleus were subjected to confocal fluorescent microscopy with a Lamp1 antibody (red) and Dapi (blue) as described in the Methods Section**. Representative images of myofibers from EDL and soleus muscle isolated from young 2 month old **(A,B)**, aged 24 month old **(C,D)**, and 2 month old Pompe mice **(E,F)** are shown. Myofibers from the EDL show two distinct Lamp1 positive structures at each pole of each nucleus whereas these structures are more dispersed and random in myofibers from the soleus muscle. Scale bar, 10μm.

White glycolytic [extensor digitorum longus (EDL)] and red oxidative (soleus) muscles were examined by confocal microscopy. Initially, analyses of fiber type specificity used myosin heavy chain markers to distinguish between white and red myofibers; however, Lamp1 immunofluorescence indicated the presence of Lamp1 positive structures at the poles of the myonuclei in 2 month-old EDL muscle fibers (Figure 1A). Although the pattern of the Lamp1 staining was more dispersed in soleus muscle fibers it remained clustered around the myonuclei (Figure 1B). After confirmation that EDL myofibers always had two distinct Lamp1 structures at each pole and soleus myofibers had a more dispersed organization, Lamp1 immunofluorescence was used to determine fiber type in all future experiments. In young healthy mice, these perinuclear organelles were similar in size to peripheral Lamp1 positive structures. However, at 24 months of age, the perinuclear Lamp1 positive structures were markedly enlarged in the EDL fibers compared to peripheral lysosomes/late endosomes (Figure 1C). Similarly, in the soleus muscle fibers the Lamp1 positive structures were also selectively enlarged when compared to peripheral structures, but they again lacked the perinuclear organization of the EDL (Figure 1D). We also compared Lamp1 staining in skeletal muscle from a mouse model for Pompe's Disease, a glycogen storage disease in which the mice present with severe muscle loss and weakness. All Lamp1 structures are significantly enlarged in Pompe's Disease, but those of perinuclear localization in both EDL and soleus muscles were larger than the peripheral structures (Figures 1E,F).

**Figure 2:**
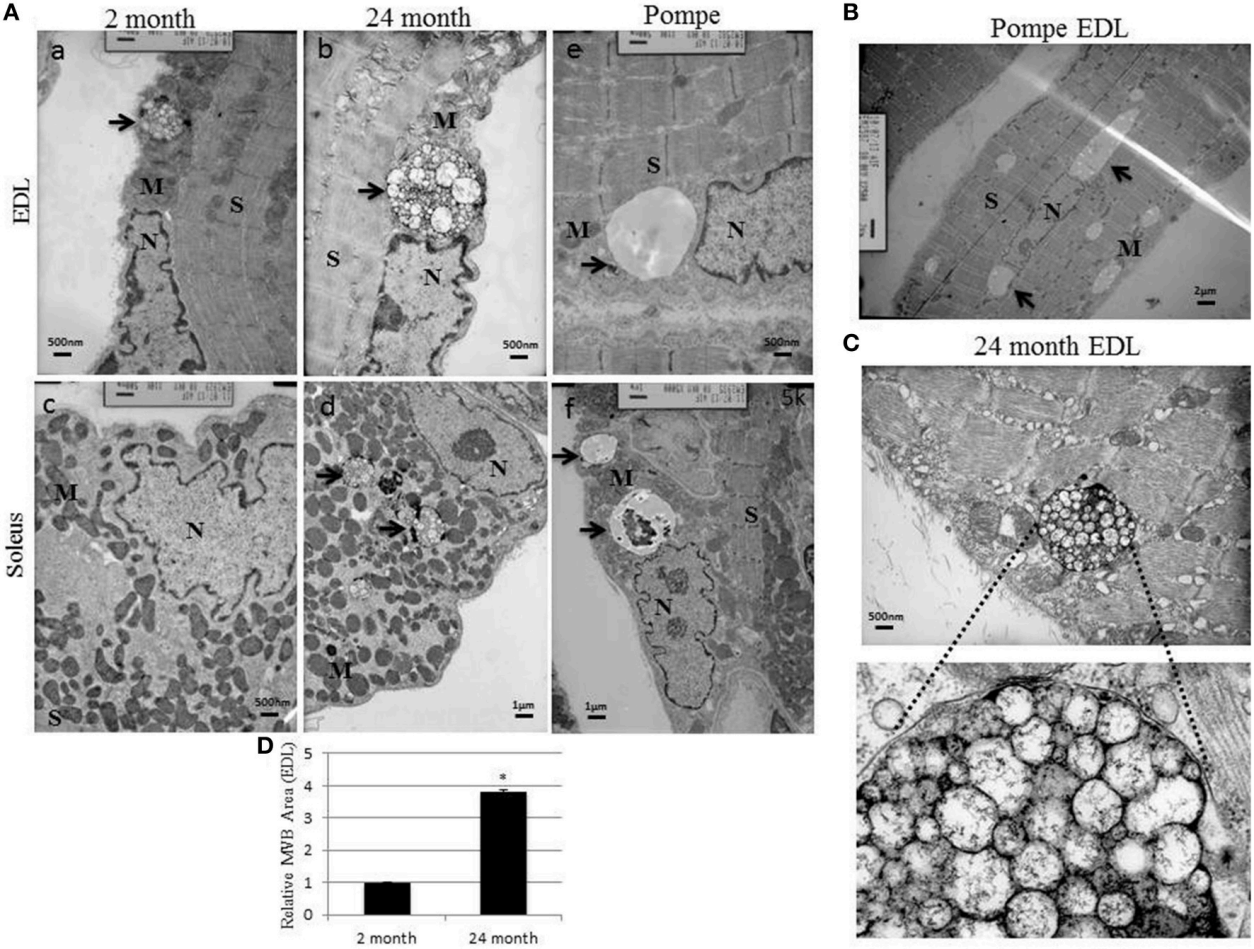
**Perinuclear structures have a multi-vesicular body (MVB) like morphology that is enlarged during muscle wasting**. Muscle sections from EDL and soleus were fixed, stained, and imaged with an electron microscope as detailed in the Methods Section. **(A)** Representative TEM images from young 2 month old, aged 24 month old, and 2 month old Pompe mice. **(B)** Low magnification representative image of the Pompe EDL myofiber. **(C)** Cropped and zoomed image of a perinuclear MVB from aged 24 month old EDL muscle. The bar in each image indicates the magnification scale size. **(D)** Quantification of the EDL perinuclear MVB size from 2 to 24 month old myofibers. The area of the organelle was measured using Image J and the graph represents the average of each group; error bars indicate SEM, *n* > 30, ^*^indicates statistical significance as assessed by *p* < 0.05. The arrow identifies the perinuclear organelle in each image. N, nucleus; S, sarcomere; M, mitochondria.

To examine these compartments at the ultrastructural level, the EDL and soleus muscles from young, aged, and Pompe mice were fixed, stained, and imaged by transmission electron microscopy. In young mice, the perinuclear structures in EDL muscle appeared to be multivesicular in nature and situated at the polar ends of the nuclei but separated by cytoplasm and other organelles like mitochondria (Figure 2Aa). Perinuclear structures were found in the EDL of aged muscle as well and they also displayed a multivesicular morphology (Figure 2Ab). The aged perinuclear organelles were also enlarged when compared to the young muscle fibers. Despite looking at many ultrathin sections of soleus muscle from young mice, we were unable to identify any multivesicular structure around the nuclei (Figure 2Ac). Multiple, small perinuclear organelles with a random organization were found in aged soleus muscle and again with a multivesicular appearance (Figure 2Ad). The same organization pattern was found in Pompe mice, but the morphology was different. In Pompe EDL, a single large organelle was found at each end of the myonuclei and they appeared to be devoid of any ILVs or osmium tetroxide staining. In Pompe soleus muscle, multiple large organelles were found around the myonuclei and appeared morphologically similar to the Pompe EDL perinuclear organelles, although some contained dark intralumenal material (Figures 2Ae,f).

In skeletal muscle from Pompe mice, these organelles can reach extremely large sizes but still retain their perinuclear organization (Figure 2B). The staining pattern of these can likely be explained by an accumulation of glycogen that does not stain with osmium tetraoxide. A cropped and enlarged close up of the aged EDL muscle shows the morphology of the perinuclear multivesicular structures (Figure 2C). The structures contain many intraluminal vesicles combined with a material that stains heavily for osmium tetraoxide (dark black stain). The average area of these perinuclear structures in white muscle was measured using ImageJ and the 24 month aged perinuclear organelles were found to be nearly 4 times as large as the 2 month organelles (Figure 2D). Individual muscle fibers from the EDL were also specifically prepared and embedded for focused ion beam/scanning electron microscopy (FIB/SEM). A cropped movie of stacked FIB/SEM images shows the full morphology of one of these perinuclear organelles (Supplementary Video S1). These structures are bundles of intraluminal vesicles encapsulated in a membrane. The FIB/SEM shows there are multiple bundles of vesicles and perhaps even multiple MVBs that make up the organelle in this perinuclear location. Again, this organelle is separated from the nucleus by cytoplasmic material and mitochondria. Together, these images demonstrate the presence of a unique Lamp1 positive perinuclear structure in skeletal muscle. These organelles have a fiber type specific organization and hypertrophy in muscle wasting phenotypes such as sarcopenia and Pompe's Disease.

### The perinuclear organelles have MVB-like qualities and store lipid

**Figure 3:**
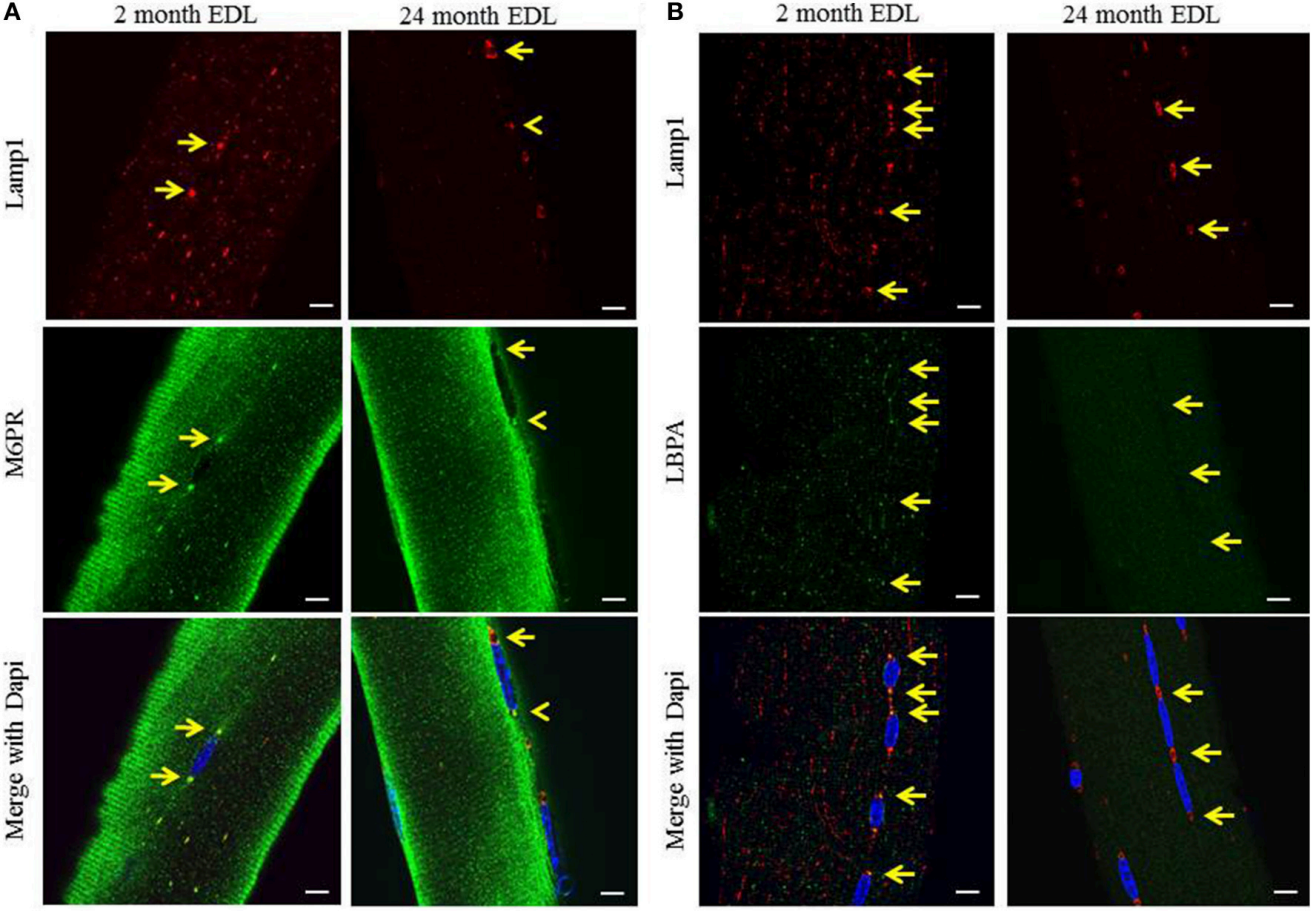
**The perinuclear structures stain for late endosome markers, which are lost upon muscle aging**. The EDL muscle was digested and myofibers were harvested and imaged by confocal fluorescent microscopy as described in the Methods Section. **(A)** Representative images showing the co-localization of the mannose-6-phosphate receptor (M6PR) late endosome/MVB marker [green], and the Lamp1 lysosome/late endosome marker [red], in young 2 month old, and aged 24 month old mice. **(B)** Representative images showing the co-localization of the specific late endosome/MVB marker, lysobisphosphatidic acid (LBPA) [green], and lysosome/late endosome marker, Lamp1 [red], in young 2 month old and aged 24 month old mice. Dapi (blue) stains the myonuclei in all images. The arrows indicate the perinuclear MVB at each pole in each image and are aligned between panels to show co-localization. Scale bar, 10 μm.

Although the TEM images clearly showed the presence of multivesicular structures, they are highly unusual when compared to electron microscopy images of other cellular MVBs. The number and density of the ILVs along with the quantity of electron dense material suggests that this is a unique MVB-like compartment. In order to determine if these Lamp1 structures have properties of classic MVBs, we examined the co-localization of the lysosome/late endosome marker, Lamp1, with markers for late endosome/MVBs. The cation-independent mannose-6-phosphate receptor (M6PR) is enriched in MVBs and lysobisphosphatidic acid (LBPA) is a unique lipid species typically enriched in MVBs. The perinuclear organelles stained positive for both markers in young EDL, consistent with these structures being MVB like (Figures 3A,B, left panels). Since the 488 nm signal intensity was greater than that of the 594 nm channel, resulting in a mostly green merged image, the yellow arrows are aligned between panels to demonstrate the co-localization of the markers at the perinuclear MVBs. The enlarged perinuclear structures in aged EDL remained positive for Lamp1 but were negative for both markers (Figures 3A,B, right panels). The overall signal intensity of LBPA in aged muscle was lower, suggesting a general depletion of the unique lipid species. Interestingly, if a perinuclear MVB remains normal size in aged muscle, it retains the MVB marker (yellow arrowhead) suggesting a correlation between the size of the organelle and the accumulation of these MVB markers. Due to high background and autofluorescence, immunostaining of Pompe fibers for MVB markers was inconclusive. Due to this and the fact that the morphology is strikingly different in the TEM images, it is not possible to unequivocally identify the structures found in Pompe myofibers as MVBs.

**Figure 4:**
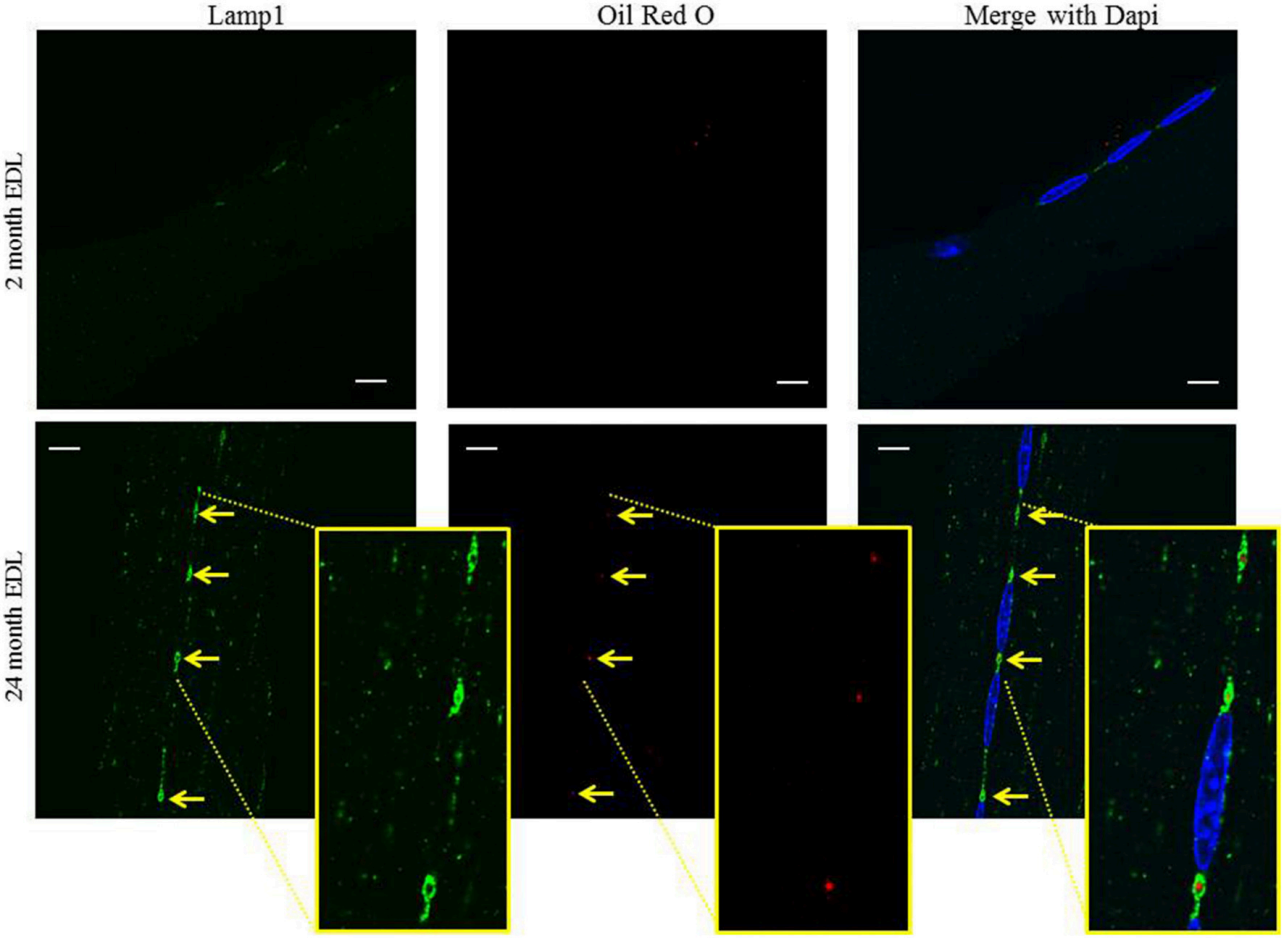
**The perinuclear MVBs hypertrophy and accumulate lipid in aged muscle**. The EDL muscle was digested and myofibers were harvested and subjected to confocal fluorescent microscopy as described in the Methods Section. The myofibers were stained with Lamp1 (green) for immunofluorescence followed by staining for oil red O (red). Dapi (blue) stains the myonuclei in all images. Representative images of the co-localization of oil red O fluorescence and the lysosome/late endosome marker, Lamp1, in young 2 month old and aged 24 month old mice are shown. The arrows indicate the perinuclear MVB at each pole in each image and are aligned between panels to show co-localization. Inserts in the bottom panel are cropped and zoomed to enable visualization of the red oil red O puncta inside the ring of Lamp1. Scale bar, 10 μm.

During aging various lipid and oxidation species have been shown to accumulate in the perinuclear regions of skeletal and cardiac muscle (Nakae et al., 2004). Oil red O (ORO) stains neutral lipids and also fluoresces so it can be used to detect lipids in conjunction with immunostaining. There is no apparent ORO staining in the young 2 month old EDL myofibers whereas, ORO staining co-localizes with Lamp1 in the aged 24 month old EDL myofibers indicated by arrows (Figure 4). Much like above, the 488 nm signal intensity is stronger than that of the 594 nm signal and the merged image does not show a yellow merged color. Cropped and zoomed inserts show the region of interest where a red dot of ORO is encompassed by a green ring of Lamp1, showing that the lipid is within these perinuclear structures. These data suggest that in skeletal muscle, there exists an unusual perinuclear MVB that hypertrophies and accumulates lipid in aged muscle.

### Perinuclear MVBs function to accumulate and store lipofuscin deposits

**Figure 5:**
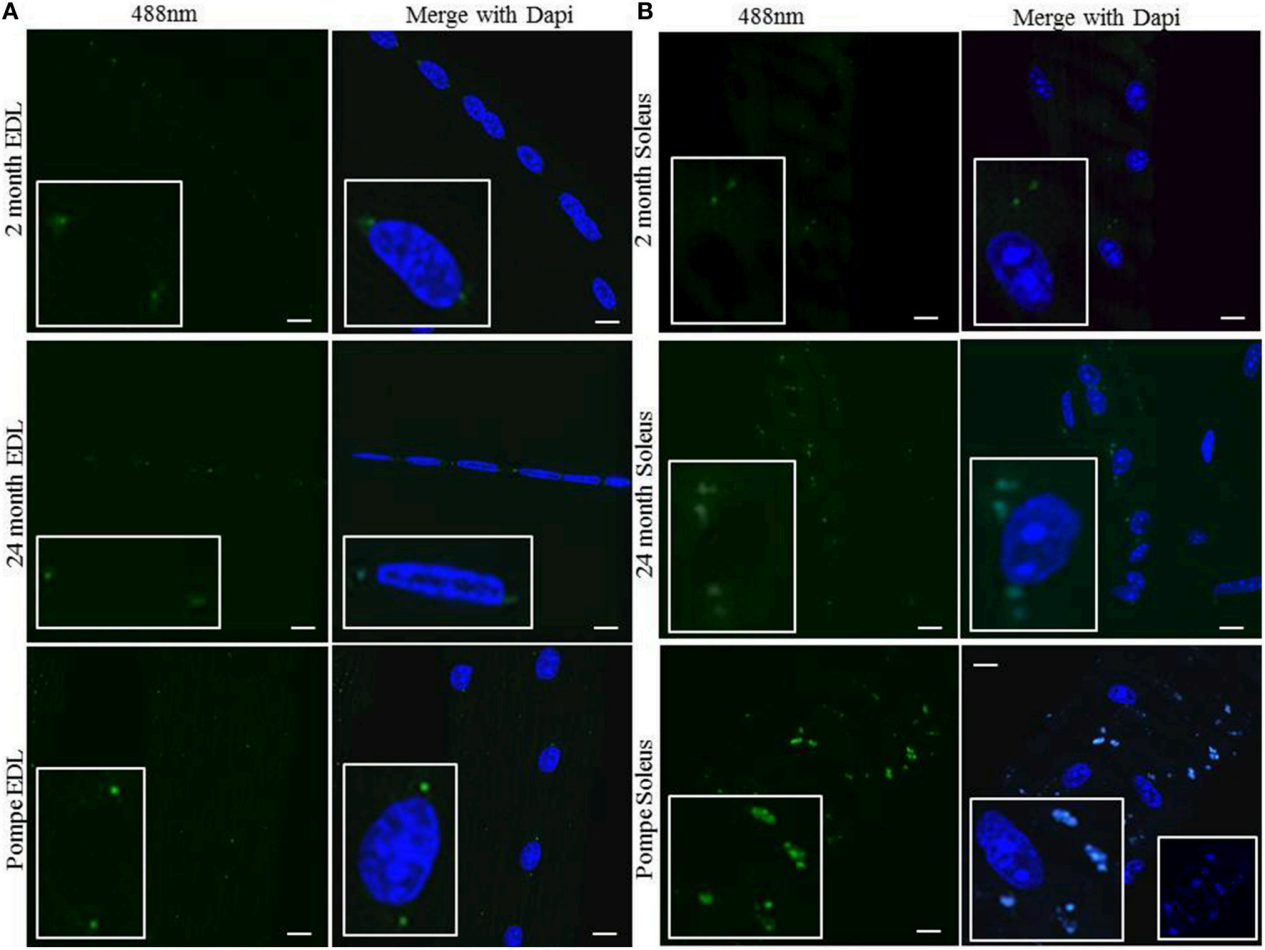
**The perinuclear MVBs contain autofluorescent lipofuscin, which accumulates upon muscle wasting**. Individual muscles were digested and myofibers were harvested from EDL and soleus muscles. Directly after fixing, the fibers were mounted on a coverslip without any immunostaining as described in the Methods Section. **(A)** Representative images of myofibers from the EDL muscle at 488 nm (green) in young 2 month old, aged 24 month old, and 2 month old Pompe mice. **(B)** Representative images of myofibers from the soleus muscle at 488 nm in young 2 month old, aged 24 month old, and 2 month old Pompe mice. Each image contains a magnified insert to show autofluorescent material around a myonucleus. The Pompe soleus sample has an additional insert of Dapi only (blue) to show the autofluorescence is strong enough to bleed into the 405 nm channel. Scale bar, 10 μm.

Lipofuscin has been well characterized as an oxidized lipid byproduct that crosslinks and accumulates in postmitotic cells. To determine whether the perinuclear MVBs contain lipofuscin we examined lipofuscin autofluorescence (Brunk and Terman, 2002; Feeney et al., 2014). In young, aged, and Pompe EDL muscle, small puncta were visualized at 488 nm that were localized to the two perinuclear MVBs in the EDL (Figure 5A). The images show only faint autofluorescence in EDL muscle that localizes to the same perinuclear areas as the MVBs. In young, aged, and Pompe red muscle, autofluorescence was visualized in and around the myonuclei (Figure 5B). In all mice, autofluorescence was stronger in the soleus than the EDL muscle, with Pompe soleus having a much stronger signal than in aged wild type mice. The signal is strong enough to bleed into the 405 nm channel, therefore appearing as blue puncta around the blue Dapi-stained myonuclei (Figure 5B, bottom right panel insert). Collectively, these data indicate that perinuclear MVBs accumulate and store lipofuscin deposits in a fiber type-specific pattern and intensity, which is augmented in diseases of muscle wasting.

### Rapamycin regulates MVB size and lipofuscin accumulation

**Figure 6:**
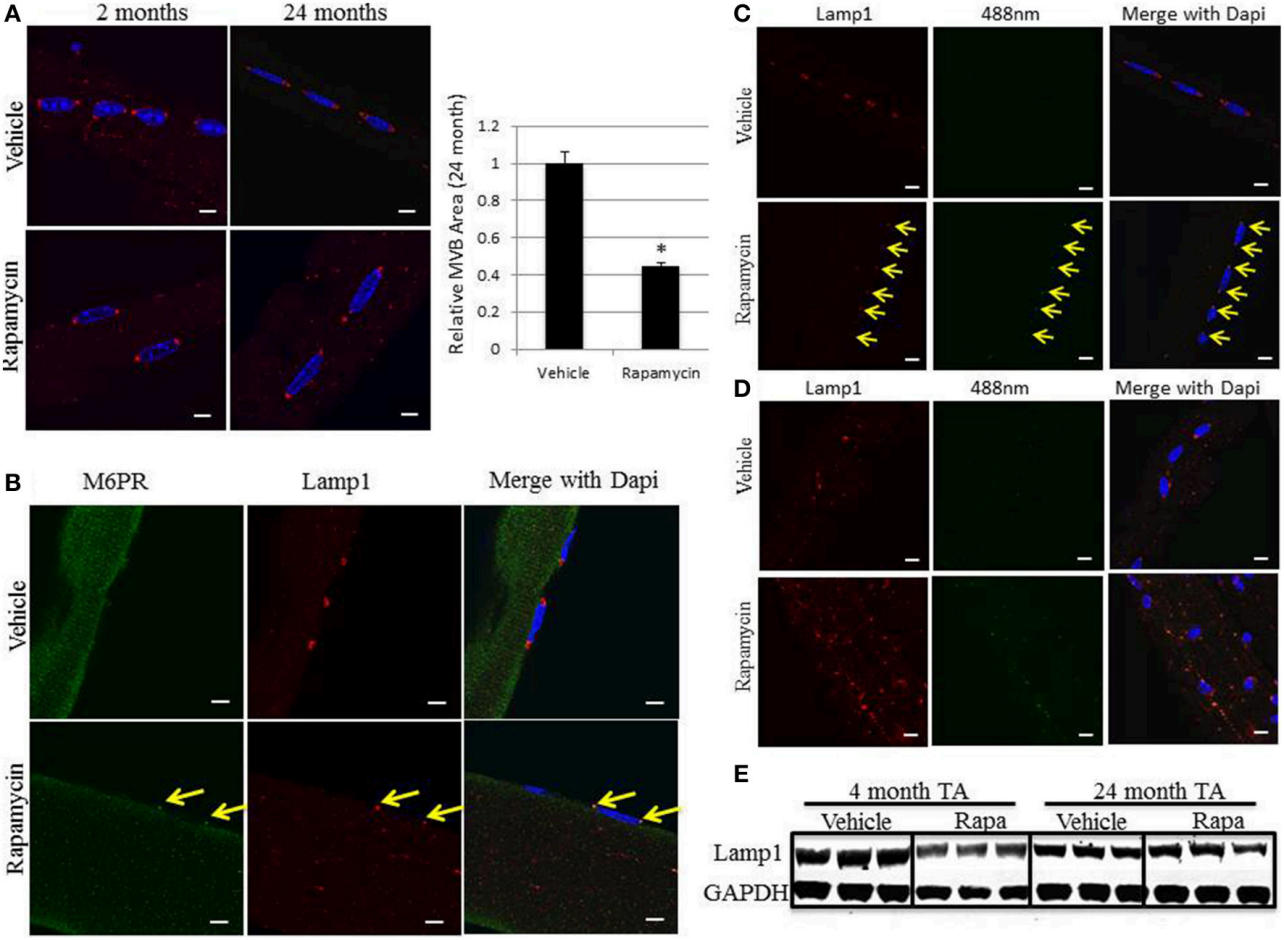
*****In vivo*** rapamycin treatment in aged mice decreases the size of the perinuclear MVBs, restores MVB markers, and increases the number and intensity of lipofuscin deposits**. Aged 24 month old mice were intraperitoneally injected with 4 mg/kg rapamycin daily for 1 month. Muscles were then dissected and individual myofibers harvested from EDL and soleus to be stained for immunofluorescence as described in the Methods Section. **(A)** Representative images of EDL myofibers immunostained for Lamp1 (red). The area of the organelle was measured using Image J and the graph represents the average of each group; error bars indicate SEM, *n* > 30, ^*^indicates statistical significance as assessed by *p* < 0.05. **(B)** Representative images of the co-localization of the M6PR late endosome/MVB marker [green], and the Lamp1 lysosome/late endosome marker [red], in aged 24 month old EDL muscle. **(C)** Representative images of EDL myofibers of autofluorescence at 488 nm (green) and Lamp1 at 594 nm (red). **(D)** Representative images of soleus myofibers of autofluorescence at 488 m and Lamp1 at 594 nm. The arrows indicate the perinuclear MVB at each pole in each image and are aligned between panels to show co-localization. Scale bar, 10 μm. **(E)** Young mice (4 month) and aged mice (24 month) were treated with and without rapamycin as above for 1 month. The tibialis anterior (TA) was isolated and tissue extracts were prepared and subjected to immunoblotting with Lamp1 and GAPDH antibodies from three independent mice per condition.

Rapamycin treatment of both aged mice and Pompe mice have shown marked improvements in muscle mass, strength, and function (Ashe et al., 2010; Zhang et al., 2014). Young 2 month and aged 24 month old mice underwent daily intraperitoneal injections of 4 mg/kg of body weight rapamycin for 1 month. The EDL and soleus muscles were dissected and myofibers isolated and immunostained. Although rapamycin treatment had no significant effect on the size or distribution of the perinuclear MVBs in 2 month old EDL muscle, there was a significant decrease in the size of the perinuclear MVBs in aged 24 month old EDL muscle. Untreated aged mice showed the enlarged perinuclear structures with a clear ring of Lamp1 and rapamycin treatment reduced these MVBs back to a single puncta of Lamp1. Using densitometry, the area of these organelles were measured and rapamycin was found to reduce the size of the perinuclear MVBs by a little more than half (Figure 6A). This reduction in size correlated with a restoration of the MVB/late endosome marker, M6PR, indicated by arrows (Figure 6B). Despite the reduction in size and restoration of M6PR co-localization, rapamycin increased the number and intensity of lipofuscin deposits in EDL muscle as assessed by examining the autofluorescence at 488 nm (Figure 6C). These lipofuscin deposits colocalize with the Lamp1 stain, showing they are in the perinuclear MVBs located at each pole of the EDL myofibers, indicated by arrows. A similar pattern was also seen in soleus muscle. Autofluorescent lipofuscin deposits are already more numerous in red muscle and rapamycin treatment increased the number and intensity of these lipofuscin deposits, which were also co-localized with Lamp1 (Figure 6D). Immunoblotting of tibialis anterior (TA) muscle extracts demonstrated no statistically significant difference in the amount of Lamp1 protein expressed between young mice (4 months) treated with and without rapamycin (Figure 6E). Similarly, there was no difference in Lamp1 levels in aged mice (24 months) treated with and without rapamycin.

## Discussion

Skeletal muscle, like other tissues, has processes in place to transport substrates around the cell and deliver them to the necessary organelles. The autophagic and endocytic pathways are examples of these complicated trafficking processes. The last step in the endocytic pathway involves the MVB, which is an organelle that packages substrates in order to deliver them to the lysosome to be degraded or for secretion via the exosome pathway (Raposo and Stoorvogel, 2013). The MVB is morphologically unique due to its ILVs, which sequester endocytic cargo and are released into the lysosomal lumen when lysosomes fuse with an MVB. While investigating these processes in skeletal muscle, unique structures with specific organizational patterns became apparent and warranted further investigation.

White and red muscle fibers have many important differences like the type of contractile proteins they express, the preferred nutrient substrates they utilize, and the relative concentration of organelles like mitochondria (Schiaffino and Reggiani, 2011; Wang and Pessin, 2013). Many papers have published fiber type specific effects and these have furthered the divide between these two types of distinct myofibers. After first analyzing these perinuclear MVB like structures, there was also a fiber type specificity in the organizational pattern. EDL muscle contained two distinct perinuclear MVBs, one at each end of the myonuclei. Aged soleus muscle contained many perinuclear MVBs that were randomly distributed near the myonuclei (Figures 1, 2). We investigated why there might be a fiber type specific pattern by staining for other organelles and cytoskeletal proteins. There was no co-localization with any other part of the autophagic or endocytic pathways and no co-localization with any other perinuclear organelle like the sarcoplasmic reticulum or Golgi apparatus. There was no difference in cytoskeletal structure after looking at microtubules, microfilaments, and intermediate filaments. The perinuclear structures did co-localize with gamma tubulin in EDL but not soleus muscle (data not shown). Thus, for at least EDL muscle, the perinuclear MVBs are located at the spindle poles of each myonuclei. Why this staining pattern differs between fiber types is the target of further investigation.

Sarcopenia is the loss of muscle mass, strength, and function attributed to aging. Interestingly, the perinuclear MVBs become enlarged in muscle from aged mice. In 24 month aged mice, the perinuclear MVBs are selectively enlarged, leaving the peripheral Lamp1 positive organelles similar in size to young skeletal muscle. This allows easy identification of this special subset of MVBs as in EDL muscle, there are two large MVBs flanking each side of the myonuclei and in soleus muscle there are many large MVBs around the myonuclei. To examine another muscle wasting disease, we looked at muscle from the mouse model of Pompe's Disease. This glycogen storage disorder causes a great excess of glycogen to accumulate, especially in the lysosome, which swells the size of these organelles. As described before, Lamp1 positive structures in Pompe muscle are greatly enlarged, yet the perinuclear MVB-like organelles are still the largest of all the structures. Some of these organelles can reach sizes equivalent of the myonuclei (Figure 2B). In addition to skeletal muscle atrophy in aging and Pompe's disease, there are several other functional and genetic muscle wasting phenotypes, for example denervation, disuse atrophy, cancer cachexia, muscular dystrophy, mTOR deficiency, and Dannon's disease to name a few. However, as far as we are aware, the presence of these unusual MVB like structures in other models of muscle wasting has yet to be examined.

The perinuclear organelles stain positive for markers of MVB/late endosomes. However, when they hypertrophy in muscle wasting phenotypes of aging and Pompe's disease, they lose these markers. This appears to be a product of the size of the organelle and not the age of the muscle, since the few small perinuclear MVBs in aged muscle remain positive for the M6PR marker whereas the large MVBs in nearby myonuclei do not (Figure 3A). This suggests that hypertrophy interferes with the endocytic trafficking of the M6PR to the enlarged MVB. In contrast, the hypertrophied perinuclear MVBs stain positive for ORO, suggesting that lipid can be delivered to the hypertrophied structures (Figure 4). The failure of MVBs in young wild type mice to show lipid accumulation could reflect a difference in delivery, but could also be due to the lack of sensitivity of the staining in the small organelles.

A particular type of lipid byproduct, lipofuscin, has been well described as a marker of aging. Lipofuscin is an amalgam of lipids, carbohydrates, proteins, and metals cross-linked by aldehydes that form a “plastic” material that cannot be further digested or undergo exocytosis. Lipofuscin accumulation is believed to be due to defects in lysosomal degradation (Brunk and Terman, 2002; Terman and Brunk, 2004). Interestingly, muscle lipofuscin has been characterized in cardiac muscle as existing at each pole of the nuclei, but no study has suggested it is encapsulated in a dynamic organelle (Nakae et al., 2004). Lipofuscin is detectable in young muscle as small puncta at each end of the white myonuclei and as randomly distributed puncta around red myonuclei, a staining pattern similar to that of Lamp1 and M6PR in these respective muscles. Aged muscle shows a marked increase in size and number of lipofuscin deposits but they still have the same organization as the perinuclear MVBs. Pompe muscle shows an even greater number, size, and intensity of lipofuscin deposits (Figures 5A,B).

An interesting pattern arises when examining these images. Red muscle consistently has lipofuscin deposits that are more intense and numerous. This may result from the large number of perinuclear MVBs in red muscle compared to the presence of only two per myonuclei in white muscle. However, the signal intensity could also be due to the metabolic needs of red muscle. This type of myofiber prefers oxidative metabolism, and is therefore using lipids as a nutrient source and creating more lipid byproducts that can eventually be cross-linked into lipofuscin. Interestingly, it has been well characterized that white muscle primarily wastes in both sarcopenia and Pompe's Disease. Red muscle is for the most part spared the loss of mass and function. This suggests that perhaps the number of deposits might not be important, whereas the size of the MVBs and lipofuscin deposits, which is greater in white muscle than red, could play a pathologic role in muscle wasting. Lastly, the TEM and FIB/SEM photos show dark black stains in the perinuclear MVBs. Lipofuscin has been shown to be osmiophilic and therefore these dark stains in the MVBs are likely the lipofuscin deposits (Figure 2C).

Young and aged mice were treated for 1 month with rapamycin and the perinuclear MVBs were analyzed. Rapamycin reduced the size of the aged MVBs, restored the M6PR marker, and increased the number and intensity of autofluorescent lipofuscin deposits (Figure 6). The first two of these results are consistent with the general benefits of rapamycin to reduce aging phenotypes (Harrison et al., 2009; Zhang et al., 2014). However, the increase in lipofuscin deposits is counterintuitive. Rapamycin inhibits mTORC1, which phosphorylates and inhibits transcription factor EB (TFEB). TFEB controls lysosome biogenesis, so rapamycin turns on TFEB, therefore increasing all the necessary factors to produce more lysosomes. The upregulation of lysosomal processes likely leads to the decrease in MVB size and the restoration of the MVB markers seen with rapamycin treatment. Interestingly, the smaller MVBs in muscle from Rapamycin-treated animals had more numerous and intense lipofuscin deposits. Previous studies have indicated lipofuscin cannot be degraded or undergo exocytotic trafficking, so it is possible that a reduction in MVB size leads to a concentration of lipofuscin and an increase in its apparent abundance (Brunk and Terman, 2002; Terman and Brunk, 2004; Jung et al., 2007). Alternatively, given the increase in M6PR staining in rapamycin-treated animals, it could reflect increased trafficking of lipofuscin to these structures. The potential role of lipofuscin in the pathology of muscle wasting and the ameliorating effects of rapamycin on sarcopenia will be addressed in further studies.

## Author contributions

BN, HZ, JB, and JP designed the experiments and BN carried out experiments. All authors analyzed the data and participated in writing the manuscript. JP and JB obtained financial support. All authors have read and approved the final manuscript.

### Conflict of interest statement

The authors declare that the research was conducted in the absence of any commercial or financial relationships that could be construed as a potential conflict of interest.

## Supplementary material

Supplementary Video S1 **FIB/SEM analysis of EDL shows a unique morphology and organization of the perinuclear MVB**. Individual myofibers from EDL muscle were harvested, fixed, and embedded in resin. The block was placed into an ultramicrotome and trimmed until the region of interest was found. Stacked images were taken with a dual beam scanning FIB/SEM and processed into a movie using Image J.
